# High accuracy label-free classification of single-cell kinetic states from holographic cytometry of human melanoma cells

**DOI:** 10.1038/s41598-017-12165-1

**Published:** 2017-09-20

**Authors:** Miroslav Hejna, Aparna Jorapur, Jun S. Song, Robert L. Judson

**Affiliations:** 1 Department of Physics, University of Illinois at Urbana-Champaign, 1110 W Green St, Urbana, IL 61801 USA; 2 Carl R. Woese Institute for Genomic Biology, University of Illinois at Urbana-Champaign, 1206 W Gregory Dr., Urbana, IL 61801 USA; 3 Helen Diller Family Comprehensive Cancer Center, University of California, San Francisco, 1450 3rd Street, Box# 3111, San Francisco, CA 94158 USA; 4 Department of Dermatology, University of California, San Francisco, 1701 Divisadero Street, San Francisco, CA 94115 USA

## Abstract

Digital holographic cytometry (DHC) permits label-free visualization of adherent cells. Dozens of cellular features can be derived from segmentation of hologram-derived images. However, the accuracy of single cell classification by these features remains limited for most applications, and lack of standardization metrics has hindered independent experimental comparison and validation. Here we identify twenty-six DHC-derived features that provide biologically independent information across a variety of mammalian cell state transitions. When trained on these features, machine-learning algorithms achieve blind single cell classification with up to 95% accuracy. Using classification accuracy to guide platform optimization, we develop methods to standardize holograms for the purpose of kinetic single cell cytometry. Applying our approach to human melanoma cells treated with a panel of cancer therapeutics, we track dynamic changes in cellular behavior and cell state over time. We provide the methods and computational tools for optimizing DHC for kinetic single adherent cell classification.

## Introduction

Many mammalian cell types, including clonal human cancer cells, can be highly dynamic in both morphology and behavior, even in homeostatic conditions. Characterizing and tracking heterogeneous behavior over time on a single cell level is critically important when studying rare events, such as the acquisition of therapeutic resistance, or transition events, such as differentiation. Live quantitative imaging with high content analysis allows for kinetic evaluation of adherent cells, but often depends on reliable fluorescent labels for accurate classification of cell state^1,2^. The disruptive and often cytotoxic effects frequently associated with fluorescent dyes and proteins can limit the length of time single cells are tracked unperturbed^3,4^. Additionally, reliable markers must be *a priori* identified to classify cell states of interest, despite observations that expression of single genes is often insufficient to predict cell state or behavior^5^. With increasing demand for kinetic quantitative classification of subpopulations within heterogeneous cultures, there is a need for reliable label-free quantitative time-lapse adherent-cell cytometry.

Digital holographic microscopy (DHM) has recently emerged as a method for visualizing mammalian cells without the use of dyes or fluorescence^6^. In DHM, one branch of a split laser beam passes through the transparent sample and recombines with the reference beam at an off-axis geometry, thereby generating interference^7^. This interference pattern (the hologram) is used to reconstruct a wavefield of the illuminated cells, which can be visualized as a three-dimensional image^8^. As the laser power is low and little energy is delivered to the cells during the process, DHM is considered non-phototoxic, permitting long-term time-lapse imaging^9–11^. DHM-derived images are quantitative, with pixel intensities proportional to the absolute phase shift of the specimen. Consequently, when phase shift images are segmented using standard approaches, dozens of cellular features related to morphology, density, and texture can be calculated for each individual cell (or other object). The measurement of cell behaviors and features from phase shift images is referred to as quantitative digital holographic cytometry (DHC).

Due to the relative affordability of commercially available DHC systems, this approach is becoming increasingly used for several applications, including cell counting, cell migration assays, monitoring for therapeutic resistance and motility characterization^12–19^. However, several challenges have hindered the more widespread adoption of this promising technology for mammalian cell biology. First, with the notable exception of the identification of cells in M-phase of the cell cycle^20–22^, the degree of single cell classification accuracy for adherent cells varies considerably between systems and significant separation is usually only achieved through comparing population averages. Further, as DHM-derived features are dependent on technical, computational, and biological variables, interpretation of these metrics must be conducted with great care. For example, optical volume has been correlated with actual cell volume, cell detachment, cell flattening, calcium fluctuations, cell cycle, cell death, cell differentiation, and protein content^8,10,23–29^. Other features are of completely unknown biological meaning. Finally, there is no established method for standardizing phase shift images for application in single cell classification. The underlying quantitative features of two visually comparable images can differ in their intensity. This discrepancy can result in datasets with similar area-based features, but divergent thickness-based features from identical cells. From a classification perspective, this is similar to identical fields of fluorescent cells imaged with two different exposure times. Whereas such dissimilarities are easily distinguished in fluorescent-based imaging using background pixel intensity, methods for standardizing DHC-derived images for single cell classification are not established. The reliability of DHM as a platform for quantitative cytometry would be increased by more standardized and accurate single cell classification.

Here we empirically define over two dozen DHC-derived features as providing biologically independent information. We use these features to train machine learning-based cell classification. We found that natural biological variation causes the cell features of homogeneous cell populations to closely follow the Gaussian distribution. We use Linear Discriminant Analysis to identify the optimal feature subspace in which the Gaussian clusters of different cell types become clearly separated and identifiable by a Gaussian Mixture Model. We find that this approach achieves high accuracy single cell separation of cell populations that are otherwise poorly distinguished using conventional DHC-derived features, and that classification of cell state by this label-free approach is comparable to both fluorescent flow and imaging cytometry. We next develop methods for optimizing DHC systems for consistent classification accuracy over long-term imaging. Finally, we demonstrate the utility of these methods with a time-lapsed screen of human melanoma cells treated with kinase inhibitors and conclude with a user workflow for standardizing phase shift images for single cell classification, validating and optimizing the reliability of classification, and conducting long-term high-accuracy label-free kinetic cell state classification.

## Results

### Machine-learning based single cell classification using DHC

**Figure 1:**
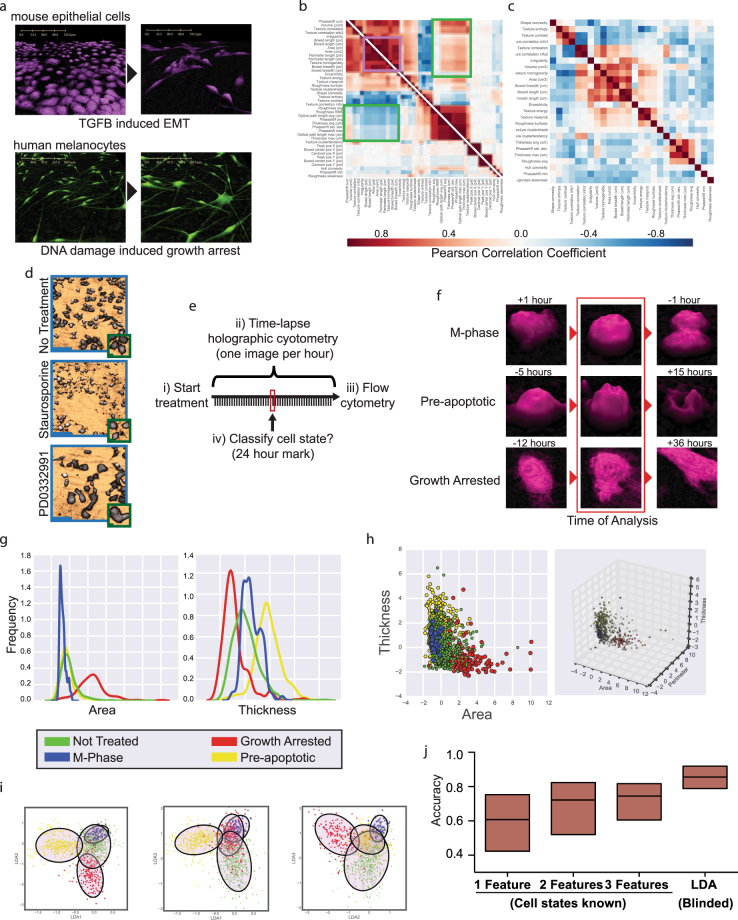
Classification of homogeneous populations. (**a**) Representative DHM images for two cell state transitions: EMT (top, NuMuG cells treated with TGFB) and DNA damage (bottom,﻿ primary human melanocytes treated with Doxorubicin). (**b**) Two split representative feature correlation matrices for mammary epithelial cells undergoing EMT (bottom left) and human melanoma cells undergoing growth arrest (top right). Areas of conserved (purple) or non-conserved (green) correlations are highlighted. (**c**) Minimal correlation matrix from thirty-five experiments, each containing ~1,000–10,000 cells. (**d**) Representative DHM images of indicated treatments. Zoomed insets show similarity of individual cells within populations. (scale bars: 100microns (blue), 10microns (green)). (**e**) Strategy for DHC-based classification using cells of verified state. (**f**) Representative images of cells designated as M-phase, pre-apoptotic, or growth arrested. Cell fates verified after the analyzed 24-hour time-point. (**g**) Distribution of area and thickness for each cell state. (**h**) 2D and 3D scatter plots of feature distribution for each cell state. (**i**) Each plane from three-dimensional LDA space (Fig. S5) derived using twenty-six features, demonstrating clusters of pre-apoptotic (yellow), growth-arrested (red), non-treated (green) or M-phase (blue) cells. (**j**) Distribution of percent accuracy of cell classification across all experiments using single, double or triple feature sets versus machine-learning based phenotypic profiling. Plots g-i used 470 pre-apoptotic, 195 growth arrested, 66 M-phase, and 1527 non-treated cells.

We reasoned that through increasing the number of independent features and conducting machine learning-based phenotypic profiling, we might more consistently separate single cell states using DHC. As all features describing objects from holographic data are calculated from the phase shift profile, it is difficult to predict *a priori* which provide unique information. To address this challenge empirically, we consolidated feature correlation data from thirty-five experiments covering a diverse cohort of morphologically distinct mouse and human cell populations: primary human melanocytes, human melanoma lines, and mouse mammary epithelial cells – each undergoing induced cell state transitions, including epithelial to mesenchymal transition (EMT), senescence, apoptosis, differentiation, and DNA-damage response (Fig. 1a). Holograms were captured using a HoloMonitor M4 DHC system^30^. The holograms were processed computationally using HStudio to produce an intensity image representing a quantitative map of light-wave phase shifts^30–32^. Individual cellular events were segmented from the resulting phase shift images. Each of the 42 optical, morphological, and positional features defined by HStudio were derived for each cell (Methods). As expected, many of the features were highly correlated, providing redundant information (Fig. 1b, purple box). However, correlations were not always preserved across transitions (Fig. 1b, green boxes). For example, cell flatness is highly correlated with cell length during EMT, but not during growth arrest. We therefore identified the minimal biological correlation for each pair of features, and combined those sets with conserved complete correlation to single metrics (Fig. 1c, Methods). The resulting 26 features provide independent information that might be used to segregate distinct cell states. Among these features were standard morphological measurements including area, perimeter, length, width, thickness, volume, convexity, irregularity, and eccentricity (circularity), each of which intuitively describes the shape of an object or cell. Interestingly, although statistically correlated, both average thickness and maximum thickness met our criteria for biological independence, suggesting that cells can display localized regions of high phase shift in addition to an overall increase in thickness. The feature list also contained a variety of texture measurements based on algorithms that calculate local variability in pixel intensity values. Eleven texture features met our criteria for biological independence and clustered into seven blocks of higher correlation. Assigning an intuitive biological interpretation to individual DHC-derived texture features is difficult, as intracellular fluctuations in phase shift could be due to either variations in the thickness of the cell surface or localized changes in optical density in the interior of the cell (for example, organelle arrangement). However, as our approach does not require a strict biological interpretation for each feature, but rather incorporates any independent measurement without bias, we retained the texture features in our further analyses.

To determine whether individual features would reliably segregate cell states, we sought to challenge the system to identify homogeneous populations of morphologically similar mammalian cells of distinct and verified cell states. The human melanoma cell line, A375, uniformly undergoes growth arrest and apoptosis when treated with the CDK4/6 inhibitor PD0332991 and the kinase inhibitor Staurosporine, respectively (Fig. S1a–e)^33^. PD0332991-treated cells failed to divide for ten days after treatment (Fig. S1a) and uniformly exhibited two markers of cellular senescence: B-galactosidase activity (Fig. S1b) and increased flow cytometric side scatter (Fig. S1c). Staurosporine-treated cells presented increased levels of cleaved CASP3 and PARP2 (Fig. S1d) and uniform surface expression of Annexin V (Fig. S1e), each an established marker of pre-apoptosis. Despite clear changes in cell state on a population level, individual cells imaged by DHM could be seen to be morphologically similar in different treatment conditions (Fig. 1d, insets). As any culture condition will contain some heterogeneity, we minimalized biological noise by tracking individual cells over a time course and then determined their fate (Fig. 1e). We first verified that 48-hours of continuous exposure to the laser resulted in no obvious cytotoxicity or morphological changes (Fig. S2). We then exposed cells to Staurosporine, PD0332991, or vehicle and acquired phase shift images every hour, a frequency that permitted observation of all cell divisions in the non-treated conditions. Cellular features were calculated for each individual cell at the 24-hour time-point based upon the segmentation data and average refractive index, and the fate of each cell included in our analyses was verified as either growth arrested (PD0332991-treated and did not divide within 24 hours of time-point), pre-apoptotic (Staurosporine-treated and underwent cell death between 10 and 24 hours after time-point), or in M phase (non-treated and divided into two cells within 1 hour after time-point) (Fig. 1f). Cell populations were then analyzed via flow cytometric assays to verify population level cell state distribution. We next asked whether individual DHC-derived features could accurately classify each of these verified cell states (Figs S1f–h and 1g). When comparing two cell states, some features achieved separation comparable or superior to flow cytometry. For example, the cell area classified growth-arrested cells with 80% accuracy and thickness separated pre-apoptotic cells from non-treated cells (Fig. S1c,e-g and Methods). However, no individual feature successfully separated all of the cell states (Figs 1g and S3). The combination of two or three features improved resolution to an average accuracy that plateaued around 68% (Figs 1h, S1h and S3). We therefore wished to determine whether inclusion of all 26 features would improve classification.

We applied a linear discriminant analysis (LDA) of the data set generated from the 24-hour holograms (Data Set 1, DS1, Fig. S4a) to project these 26 features onto the three-dimensional space that best separates the untreated, actively dividing, growth-arrested and apoptotic cell states. The resulting LDA plot clearly shows 4 clusters that can be separated by an unsupervised Gaussian Mixture Model without prior knowledge of the cell states (Figs 1i and S5). The four observed cell populations are classified with greater than 90% average accuracy, in some cases reaching 100% population separation. Using two separate DHC set-ups, we then repeated the experiment ten additional times to generate independent data sets without manual verification of individual cell state (Fig. S4a). These data sets (DS2–11) contain biological heterogeneity, and represent a realistic and tractable experimental set-up for most applications. With these data sets, the clear separation into distinct clusters was again not possible using only sets of one to three features, but the LDA predictions reliably increased the accuracy of cell classification by 22%, to an average of 85% (Fig. 1j). Importantly, classifiers trained on sets of three independent experiments retained high accuracies when applied to the remaining independent experiments (Figs S1i and S4b). To validate the robustness of this workflow, we trained a new classifier on the combined data from these ten experiments (DS2–11) and applied it to independent data sets generated with a third DHC set-up (DS12–15, Fig. S4). We again observed high classification accuracy (Fig. S6a,b). We further generated four data sets using cells grown in different growth medias (DS18–21) and found our strategy continued to classify cell states with high accuracies (Fig. S6c). As the generation of data sets spanned over ten cellular passages, separate batches of media, months, and distinct DHC set-ups, these observations indicate that the integrity of the DHC-derived classifier withstands standard biological and technical fluctuations.

To compare classification using DHC-derived features to more established forms of cytometry, we treated A375 cells with five compounds with known effects on cell cycle arrest, cell death, or apoptosis. After 24 hours, wells were first imaged and analyzed with the previously trained DHC-derived classifier (Fig. S4a, DS16, Fig. S4b, Classifier 3, and Fig. S7a). Subsequently, wells were analyzed using either fluorescent flow cytometry or fluorescent imaging cytometry. Flow cytometric assays included Annexin-V staining for early apoptosis (Fig. S1e), fixed-cell propidium iodide (PI) staining for cell cycle (Fig. S7b), and live-cell PI staining for cell death (Fig. S7c). Imaging cytometric assays included Ki-67 staining for cellular proliferation and cleaved CASP3 staining for apoptosis (Fig. S7d). The classification of pre-apoptotic cells by DHC clustered with the other cytometric assays measuring apoptosis and cell death (Fig. S7e). The most correlated analysis was cleaved CASP3 detection by imaging cytometry. Similarly, classification of growth arrested cells by DHC clustered with the other cytometric assays for cell cycle arrest. These analyses demonstrate that DHC-derived features are a reliable basis for label-free single cell classification of adherent mammalian cells and can be used both independent of and complementary to traditional forms of cytometry.

### Development of standardization metrics of DHC-derived features

**Figure 2:**
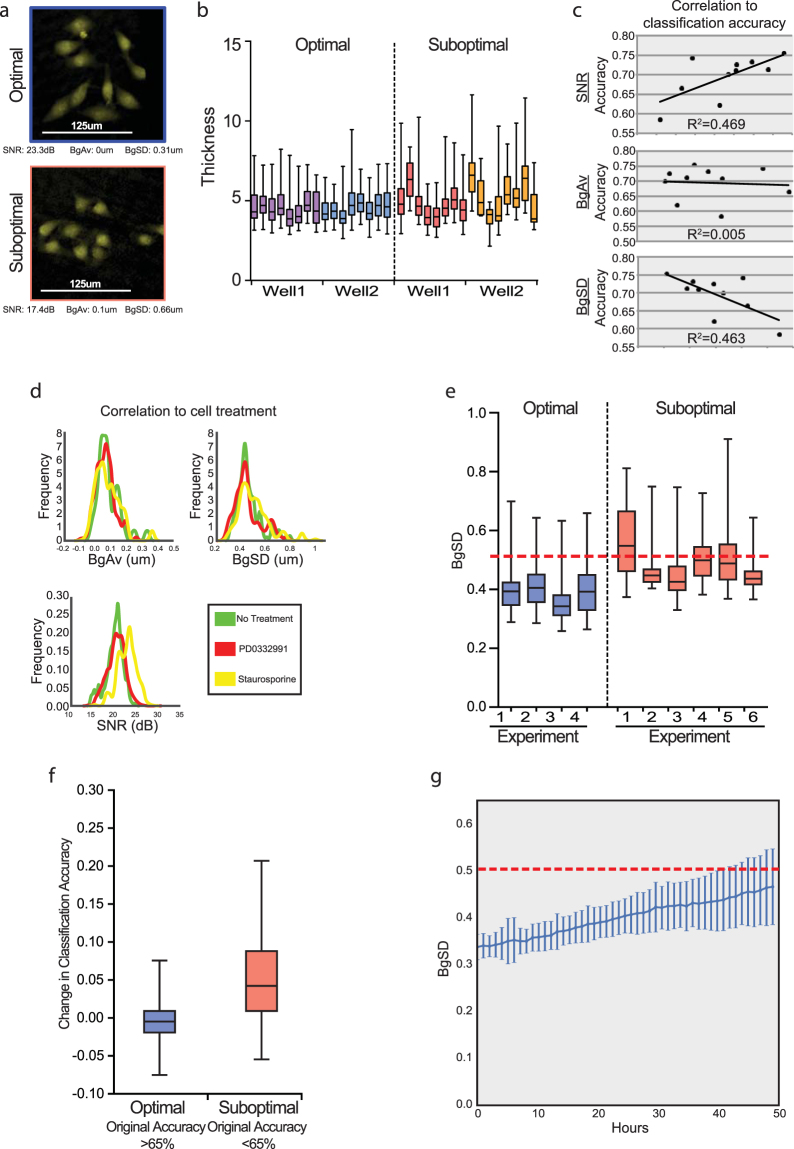
Development of standardization metrics for time-lapse classification. (**a**) Representative DHM images of cells taken with optimal or suboptimal calibration. (**b**) Distribution of cellular thickness across individual holograms taken from the same well. Visually similar images yield diverse quantities. (**c**) Correlation of potential standardization metrics (horizontal) with classification accuracy (vertical). (**d**) Distribution of potential standardization metrics segregated by cell state. (**e**) BgSD for ten experiments conducted with different calibrations. Dotted red line indicates BgSD threshold. (**f**) Classification accuracy improvement after controlling for BgSD in experiments from e. (**g**) BgSD over time from 49 image series taken continuously without calibration across 17 different experiments (standard deviation of mean). Dotted red line indicates BgSD threshold. Plots c-f are based on ~24,000 cells across 10 experiments.

We next sought to characterize the accuracy of DHC classification for long-term time-lapse experiments using the aforementioned LDA model trained on the 24-hour data (DS1). We reasoned that imaging every hour and tracking individual cells provided two distinct advantages over other forms of cytometry. First, by running an LDA classification on the same cells every hour, errors in any individual classification (here, expected to be approximately 10–15% on average) could be reduced by serial re-classifications. Second, this approach would allow for the kinetic classification of individual cells – monitoring changes in cell state and its relationship to division and motility. However, the lack of a standardizing metric for phase shift images prevents confident interpretation of changes in DHC-derived features over time. As in any laser-based analytical method, the diodes widely used in DHM platforms require calibration prior to each experiment, and become increasingly suboptimal as time passes. The resulting phase shift images are visually similar, but the derived quantitative data vary significantly (Fig. 2a,b). As the time from the last calibration increases, it is difficult to distinguish whether changes in feature values represent actual changes in cell state or drifting measurements. We reasoned that traditional image quality control metrics such as signal-to-noise ratio (SNR) or average background signal (BgAv) (Fig. S8) might bin phase shift images into groups containing comparable quantitative data. However, upon testing a range of calibrations, we found that BgAv did not correlate with the accuracy of classification (Fig. 2c) and SNR was not independent of the state of cells being imaged (Fig. 2d). Interestingly, we noticed that the degree of fluctuation of BgAv (BgSD) varied considerably between phase shift images (Fig. 2e). BgSD was not influenced by cell state and was predictive of classification accuracy (Fig. 2c,d). To determine whether categorization of holograms based upon BgSD increased the accuracy of classification, we removed holograms with BgSD >0.5 (Fig. 2e, red line and Methods). Despite the consequential decrease in the number of data points, this filtering strategy selectively increased the classification accuracy in experiments containing suboptimal holograms by an average of 5%, but had no effect when most images passed the threshold (Fig. 2f). Taking low BgSD as a proxy for high classification accuracy, we determined how long a calibrated system continues to capture quantitatively comparable holograms during a time-lapse experiment. We collected 49 time-series, acquiring holograms and BgSD every hour for two days per experiment. As expected, BgSD drifted upward over time, but remained below the quality threshold for 40 hours (Fig. 2g). These data provide a metric for assessing the duration a calibrated laser module will provide high accuracy LDA-based classification. These studies further identify BgSD as a useful metric for identifying phase shift images harboring comparable quantitative data.

### Deep screening of kinase inhibitors

**Figure 3:**
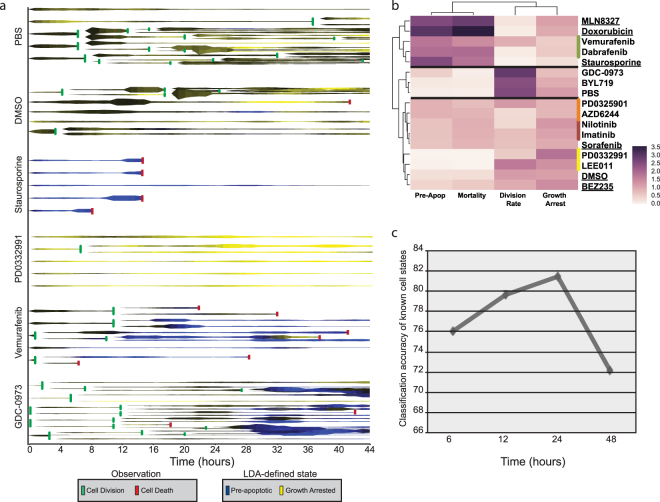
Kinetic high depth screen of kinase inhibitors. (**a**) Example rocket plots of five representative cells over six conditions. Each horizontal line represents a single cell over time, where thickness correlates with microns moved since previous hour, colorimetric represents confidence of cell state based on morphology, and horizontal bars represent cell division or death. (**b**) Heatmap depicting clustering of compounds by four super-metrics derived from rocket plots, normalized to the mean value of all conditions (see Methods). Targeted BRAF compounds (green), MEK inhibitors (orange), Bcr-Abl (red) and cell cycle inhibitors (yellow) are highlighted. Compounds dissolved in DMSO are underlined. Based on observation of 775 cells across 17 different treatment conditions. (**c**) Average accuracy of control conditions (Staurosporine, PD0332991, and not treated) at indicated time points. Accuracy peaks at 24 hours (the time-point the experiments used to train the classifier were captured) and drops after 40 hours.

We next tested the capacity of our platform for long-term kinetic deep screening by treating A375 human melanoma cells with a panel of well-characterized kinase inhibitors known to have toxic, growth-arresting or negligible effects. Growth curves were generated by counting the total number of cells in each series of phase shift images every four hours (Fig. S9a). On a population level, the effect of the compounds on cell proliferation matched previous observations. The targeted BRAF^V600E^ inhibitors Dabrafenib and Vemurafenib were acutely toxic along with the apoptosis-inducing compounds, Staurosporine and Doxorubicin. Other inhibitors of MAPK signaling were less toxic, whereas regulators of cell cycle slowed growth, and PI3K and BCR-ABL kinase inhibitors exhibited no population level effect. To investigate additional information provided by DHC, we monitored total cell movement, cell divisions, cell death and the 26 biologically independent features each hour for at least 40 cells per condition (DS17). Using the classifier we trained on previous experiments (Fig. S4, Classifier 3), we assigned a probability at each time point that a cell was pre-apoptotic or undergoing CDK4/6 inhibition. These probabilities, along with their relationships to cell division, cell death, and cell motility were visualized using “rocket” plots for each cell (Fig. 3a). We observed a clear association between cells predicted to be pre-apoptotic and eventual cell death. This state was associated with targeted BRAF^V600E^ inhibitors, apoptosis-inducing compounds, and MLN8347, consistent with the recent discoveries that AUORA kinase inhibitors are effective agents against BRAF^V600E^-driven melanoma cell lines (Fig. S9b)^34,35^. The morphological state associated with CDK4/6 inhibition was cyclical in normal dividing cells, but sustained in growth-arrested cells. We further observed that while the response of each individual cell was uniform for some compounds, other inhibitors elicited variable responses. For example, GDC-0973 induced growth arrest in some cells but hyper-proliferation followed by apoptosis in others (Fig. 3a). We quantitated this cell-to-cell response heterogeneity by clustering the cell-level data contained within the rocket plots (Fig. S9b) and further clustered each treatment by apoptosis, arrest, mortality, and division rate (Fig. 3b and Methods). This provided a more detailed classification of the screened compounds, successfully clustering the targeted compounds from other acutely toxic compounds and compounds that induce growth arrest. Interestingly, the less acutely toxic compounds were grouped by compound vehicle used (either DMSO or PBS), indicating that the platform has the sensitivity to differentiate between the subtle effects of commonly used solvents within 48 hours of treatment. Within the DMSO cluster, inhibitors targeting the same kinase grouped together, demonstrating the platform can classify compounds by molecular function. Finally, we tracked individual cells through the three-dimensional LDA space. Although the accuracy of identifying Staurosporine and CDK4/6 inhibitor treated cells remained high (>70%) throughout the experiment, accuracy peaked at 24-hours, consistent with the fact that the classifier we used was trained on static time points of cells treated for 24-hours (Fig. 3c). These data demonstrate that our platform captures the dynamic response of these cells to the inhibitors, successfully identifying not only the treatment, but also the duration of treatment, with single cell resolution.

## Discussion

We have developed methods for robust and kinetic label-free classification of single adherent cells into functional states. When analyzing more than two states, accurate single cell classification required a greater separation of functional states than what was achievable using standard DHC-derived features. We attained the necessary separation through three advances in methodology. First, we empirically identified DHC-derived features that provided independent information for phenotype profiling. Second, we used these features to train a machine-learning classifier. It is important to note that with this approach, it is not necessary to infer the biological meaning of any single feature. Third, we identify a metric for comparing quantitative data across phase shift images, which permitted us to optimize our datasets specifically for images conducive to high classification accuracy.

Three recent reports have used machine-learning approaches for similar label-free cell classification. Yoon and colleagues achieved similarly high accuracy classification of lymphocytes in suspension using features from three-dimensional refractive index tomograms^36^. Similarly, Roitshtain and colleagues reported high accuracy classification of different melanoma cell lines in suspension using 15 DHC-derived features^37^. Both reports demonstrate high accuracies of machine-learning driven classification using label-free features measured at single time-points of cells in suspension. Our data complement these observations by reporting a method that permits similar label-free techniques for continuous monitoring of adherent mammalian cells undergoing dynamic cell state transitions over time. More similar to our approach, Molder and colleagues captured full-field phase shift images of adherent mammalian cells undergoing a single cell state transition^38^. In this study, machine-learning classifiers using just six features similarly outperformed classification with single features. Interestingly, despite more pronounced morphological changes and a more advanced segmentation approach, classification accuracy was lower and more varied compared to the cell states analyzed here. We expect that combining the advanced segmentation techniques applied by Molder and colleagues with the expanded set of independent features and hologram standardization techniques shown here will increase the classification performance of both approaches.

**Figure 4:**
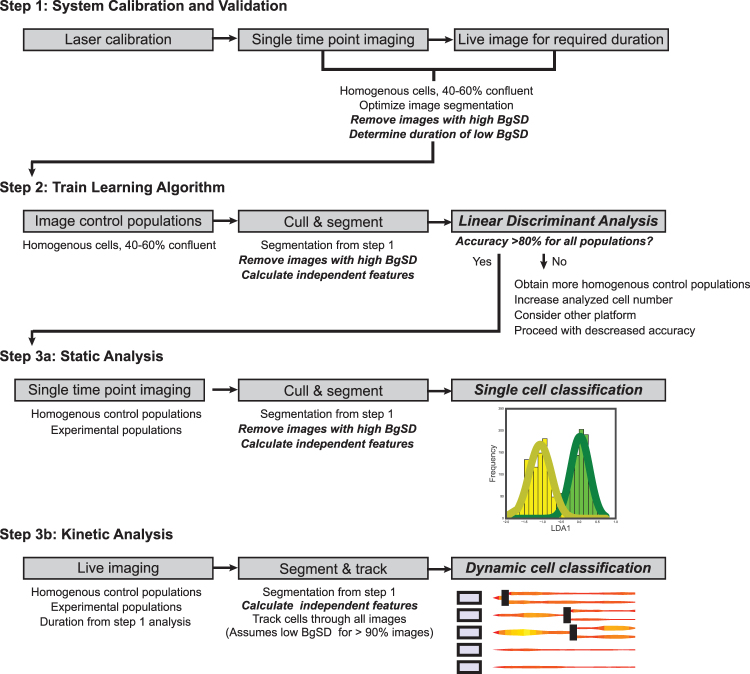
Methods and tools for conducting single cell classification with DHC. Step 1 is recommended for validating and optimizing a DHM platform prior to classification. Of importance is determining the expected rate of high BgSD images and changes of this rate over time. Step 2 is used to train a classifier to determine expected optimal accuracy. Static or kinetic experiments are then conducted with these parameters, known accuracies and known limitations. Italicized text describes methods developed here or code available upon request.

The basic technical set-up for DHM is relatively simple as compared to fluorescent microscopy and is consequently a more affordable option for phenotypic profiling. The system used in this study, the HoloMonitor M4, can be assembled inside a standard mammalian tissue culture incubator^30,39^. We provide a workflow and access to code for other groups to establish similar methods using comparable DHC platforms (Fig. 4). Although our analyses were limited to M4 DHC set-ups, we hope that the quality control metrics developed here will serve to standardize phase shift images used for quantification and allow greater comparison of data across studies and platforms. We emphasize that successful classification by DHC, like other forms of cytometry, requires, first, calibration of the system for the purposes of classification (we suggest BgSD), and second, as homogeneous of control populations as possible. The outlined strategy can be used for either static classification (investigating the percent of a population in a defined state) or kinetic classification (investigating changes in state over time). We demonstrate that this analysis is non-toxic such that the cells can be further analyzed with complementary cytometric or molecular assays once the classification is complete.

We foresee a variety of uses for this platform. With the recent advent of CRISPR-Cas9 mediated genome engineering, the genomes of primary human cell lines can now be edited with high efficiency. This platform would allow for the direct comparison of edited cells to isogenic sibling control cells. Other uses include the identification and functional characterization of rare subpopulations of cells, or of transient cell populations within extensive differentiation protocols, without the need of known markers.

## Methods

### Cell culture

The A375 human melanoma cell line was obtained from the Bastian lab (UCSF) and cultured in RPMI-1640 without HEPES supplemented with 10% FBS (Corning, 35-010-CV), 0.1 mg/mL Pen-Strep, and 0.002 mg/mL L-Glutamine. The cell line was validated based upon driver mutation (BRAF^V600E^ and CDKN2A) status. The NuMuMg mouse mammary cell line was obtained from the Derynk lab (UCSF) and cultured in DMEM H-21 supplemented with 10% FBS, 0.1 mg/mL Pen-Strep, 0.002 mg/mL L-Glutamine and 0.2 mg/mL insulin (Sigma, 91077 C). The cell line had the expected epithelial morphology and EMT response to TGFB, but is otherwise un-validated in our hands. Normal human melanocytes were purchased directly from ThermoFisher (C0245C), and cultured in Melanocyte medium (ThermoFisher, M254500) supplemented with HMGS (ThermoFisher, S0025). All lines in the laboratory, including those used during these studies, are verified routinely as mycoplasma negative using the ATCC Mycoplasma Detection Kit (30–1012-K).

For each holographic imaging experiment, passage-matched A375 cells were first split from wells of defined density (75% confluence) into 6-well culture plates (Sarsdedt, 83.3920) at 50,000 cells per well. NuMuMg cells and normal human melanocytes were seeded at 20,000 and 80,000 cells/well, respectively. After 24 hours, the media was replaced with 3mLs fresh media containing diluted small molecules and ligands at the concentrations shown in Table 1. Aseptic optical lids (HoloLids, Phase Holographic Imaging) were placed on each plate to prevent condensation and surface vibrations to allow for 36 to 52 hours of time-lapse imaging.

**Table 1:** Concentrations of the small molecules and ligands used in holographic imaging experiments.

Drug	Company	Cat. No.	Working conc
PD0332991	Selleckchem	S1116	1000 nM
Staurosporine	Tocris Bioscience	1285	1 nM
TGF-Beta	R&D Biosystem	240-B-002	0.2 ng/ml
Doxorubicin	Fischer	BP25161	10 nM
Trametinib	Selleckchem	S2673	1 nM
Cobimetinib	Selleckchem	S8041	0.84 nM
Alpelisib	Selleckchem	S2814	100 nM
Ribociclib	Selleckchem	S7440	61.4 nM
Vemurafenib	Selleckchem	S1267	10 nM
Dabrafenib	Selleckchem	S2807	0.16 nM
Sorafenib	Selleckchem	S8599	10 nM
PD0325901	Selleckchem	S1036	0.1 nM
MLN8327	Selleckchem	S1133	100 nM
Selumetinib	Selleckchem	S1008	2 nM
Imatinib	Selleckchem	S2475	200 nM
Nilotinib	LC Labs	N-8207	10 nM
PBS	UCSF-CCF		0.01%
DMSO	Sigma	472301	0.01%

For cell state verification and cytometric analysis, cells were either: i) stained using a Senescence B-Galactosidase kit (Cell Signaling, 9860) and imaged using a Lieca Dmi1 light microscope; ii) analyzed for changes in side-scatter using a BD FACSCalibur; iii) lysed for Western analysis using RIPA Lysis and Extraction Buffer (ThermoFisher 89900) supplemented with Halt Protease Inhibitor cocktail (ThermoFisher 87786); iv) stained in suspension with a Pacific Blue tagged Annexin V antibody (Biolegend 640917) or propidium iodide (ab14083) for 20 minutes before FACS analysis; v) fixed in 4% PFA for 15 minutes then stained for Ki-67 (558615) or cleaved CASP-3 (9661) and imaged using (ThermoFisher, EVOS FL); or vi) ethanol (70%) fixed, RNAse treated (R-6513) and stained with propidium iodide for 30 minutes before FACS analysis.

### Molecular Biology

Protein extracts were mixed with NuPAGE LDS Sample Buffer (ThermoFisher NP0007), NuPAGE Sample Reducing Agent (ThermoFisher NP0004) and heated for 10 min at 70 °C. Protein (10ug/lane) was resolved on NuPAGE Novex 4–12% Bis-Tris Protein Gels, 1.0 mm, 15-well (ThermoFisher NP0323BOX) and transferred using a Mini-Trans-Blot Cell onto 0.22Um PVDF membranes (ThermoFisher 88520). Membranes were blocked with Membrane Blocking Solution (ThermoFisher 000105) for 1 hour at room temperature (22 °C). The membranes were then incubated overnight at 4 °C with primary antibodies at the following dilutions: anti-Caspase 3 (CST 9661) 1:1000, anti-PARP2 (CST 9542) 1:1000, anti-Actin (CST 4970) 1:5000. The membranes were then washed four times with PBS and 0.5% Tween20 (TBST) and incubated with horseradish peroxidase-conjugated secondary antibody (anti-rabbit NA934) 1:15000 for 1 h at room temperature. The membranes were then washed four times with TBST and were visualized with Lumina Forte Western HRP substrate (Millipore WBLUF0500).

### Digital holographic microscopy and analysis

Digital holographic microscopy was performed using HoloMonitor M4 imaging cytometers with high precision automated stages (Phase Holographic Imaging, Lund, Sweden) in which the object beam and reference beam of 50:50 split 635 nm laser light are each, respectively, either passed through the sample then collected by a PLN 20x objective (Olympus) or tilted to create an off-axis configuration, before being directed to a 1.3MP CMOS global shutter USB 2.0 camera^30^. Wavefields were reconstructed by HStudio using a combination of the basic Fresnel transform and angular spectrum methods, as previously reported^31,32^. Absolute thickness was estimated by approximating and holding constant the average difference in the refractive index between cells and media as 0.04.

Holograms of A375 cells were generated in a standard mammalian cell culture incubator at 37 °C and 5% CO_2_. DHM images were generated and segmented using the HStudio (v2.6.3). The software calculates the most well focused image from the range of potential focal distances. DHM images were uniformly segmented using an Otsu’s method algorithm with an optical thickness threshold of 130 and minimal object size of 26. Optical metrics in the non-segmented space (Figs 2 and S8) were calculated as follows:

1 $${\rm{BgAv}}={{\rm{\Sigma }}\mathrm{PS}}^{{\rm{BG}}}{/\mathrm{CT}}^{{\rm{BG}}}$$

2 $${\rm{BgSD}}={[{\rm{\Sigma }}{({{\rm{PS}}}^{{\rm{BG}}}-{\rm{BgAv}})}^{{\rm{2}}}/({{\rm{CT}}}^{{\rm{BG}}})]}^{1/2}$$

3 $${\rm{SNR}}={\rm{20}}\,\ast \,{\mathrm{log}}_{{\rm{10}}}\{[({{\rm{\Sigma }}\mathrm{PS}}^{{\rm{OB}}}/{{\rm{CT}}}^{{\rm{OB}}})-{\rm{BgAv}}]/{\rm{BgSD}}\}$$

Where PS is the phase shift value of a pixel. CT is the pixel count. Superscript BG refers to all pixels not included in segmented objects. Superscript OB refers to all pixels included in segmented objects.

### Correlation matrices

Pearson correlation coefficient was used to assess the biological independence of each pair of features generated by HStudio. Since the correlation between features depends on the biological state of cells and the induced transition (Fig. 1b), the smallest correlation coefficient (in absolute size) among 35 experiments measuring cell transitions between different states under different treatments was plotted in Fig. 1c to evaluate the biological independence of each feature pair. Of 42 variable cell metrics provided by HStudio, we eliminated features that have more than 98% correlation in all 35 experiments. We also eliminated features that were a constant multiple of another feature already used and metrics that measure the position of a cell within the image. The resulting list of non-redundant features used for classification of cell states is as follows:

Area (μm^2^), Boxed breadth (μm), Boxed length (μm), Eccentricity, Hull convexity, Irregularity, Perimeter length (μm), Phaseshift min, Phaseshift std. dev., Roughness avg, Roughness kurtosis, Roughness skewness, Shape convexity, Texture clustershade, Texture clustertendency, Texture contrast, Texture correlation, Texture correlation info1, Texture correlation info2, Texture energy, Texture entropy, Texture homogeneity, Texture maxprob, Thickness avg (μm), Thickness max (μm), Volume (μm^3^).

### Cell state classification

Linear discriminant analysis was used to project the cell features into a two or three-dimensional linear subspace that separates the different treatment categories of cell states (Fig. S5a,b and Supplemental Text). A Gaussian mixture model was then fitted to the projected states in order to assign class probabilities to regions corresponding to the different cell states (Fig. S5c,d). We bootstrap sampled 1000 cells from each treatment group in order to have cell state clusters of comparable size in the training set.

When considering only commonly used DHC-derived features (T, P, A, or V), clusters corresponding to different cell states were poorly separated; thus, unbiased Gaussian mixture model fitting did not yield a useful classification in this case. Instead, in order to assess the most favorable classification accuracy under Gaussian models, we fitted a Gaussian model to each condition separately (supervised learning) and used the resulting mixture model to predict cell states (Fig. 1g,h,j).

### Hologram Filtering

To determine whether removing the holograms with high values of BgSd improved the classification accuracy, incrementally increasing values of the thresholds (0.45, 0.5, 0.55, 0.6) were set and the average classification accuracy across ten replicate experiments (4 with optimal calibration and 6 with suboptimal calibration) were evaluated (Fig. 2e,f). The optimal threshold value of BgSd that lead to the highest average classification accuracy is BgStd = 0.5. With suboptimal calibration, filtering the holograms lead to the average removal of 40% of holograms and 5% average increase in classification accuracy. With optimal calibration, on the other hand, only 10% of holograms were removed and the increase in classification accuracy was less than 1%.

### Single cell analysis

Each cell was tracked throughout a 48 h time frame with measurement performed every hour. At each time point, the cell state classification model was used to assign to each cell a probability of three states (growth arrested (CDKi), pre-apoptotic (St) or neither). For display purposes, probabilities were smoothed with Savitzky-Golay filter with degree 2 and window 5 (Fig. 3a). In order to better understand the overall response of cells to treatment, cell behavior was condensed into 6 biologically relevant super-metrics for population analysis: pre-apoptotic indicator, growth arrested indicator, division rate, mortality, motility, and variability of response (Fig. S9b). The pre-apoptotic indicator is the probability of a cell within the population being in the St state and set to 1 upon cell death. Growth arrested indicator is the probability of a cell within the population being in the CDKi state. Division rate is the probability of a cell within the population dividing. Mortality is the ratio of dead to all cells at the last time point. Motility is the probability that a cell within a population is undergoing a period of hypermotility. Variability of response is defined as the average variance of the predicted morphology-defined state, division rate and motility rate between the different cells in the experiment. We normalized the super-metrics to have the average value across all cell conditions to be 1. Hierarchical clustering of the drugs was performed using Euclidean distance and average linkage (Fig. 3b).

### Code availability

A python code used for our analysis is available upon request. Code for the LDA analysis is provided in Supplemental Text 1.

## Supplementary information

Supplementary information
