# Supplementary material for: High accuracy label-free classification of single-cell kinetic states from holographic cytometry of human melanoma cells
Source: Sci Rep. 2017 Sep 20;7:11943. doi: 10.1038/s41598-017-12165-1 (PMC5607248; doi:10.1038/s41598-017-12165-1)
Supplement: Supplementary file 1 — Supplementary information [file 41598_2017_12165_MOESM1_ESM.pdf]

High accuracy label-free classification of single-cell kinetic states from holographic cytometry of human melanoma cells

Miroslav Hejna, Aparna Jorapur, Jun S. Song, Robert L. Judson

Supplemental Material

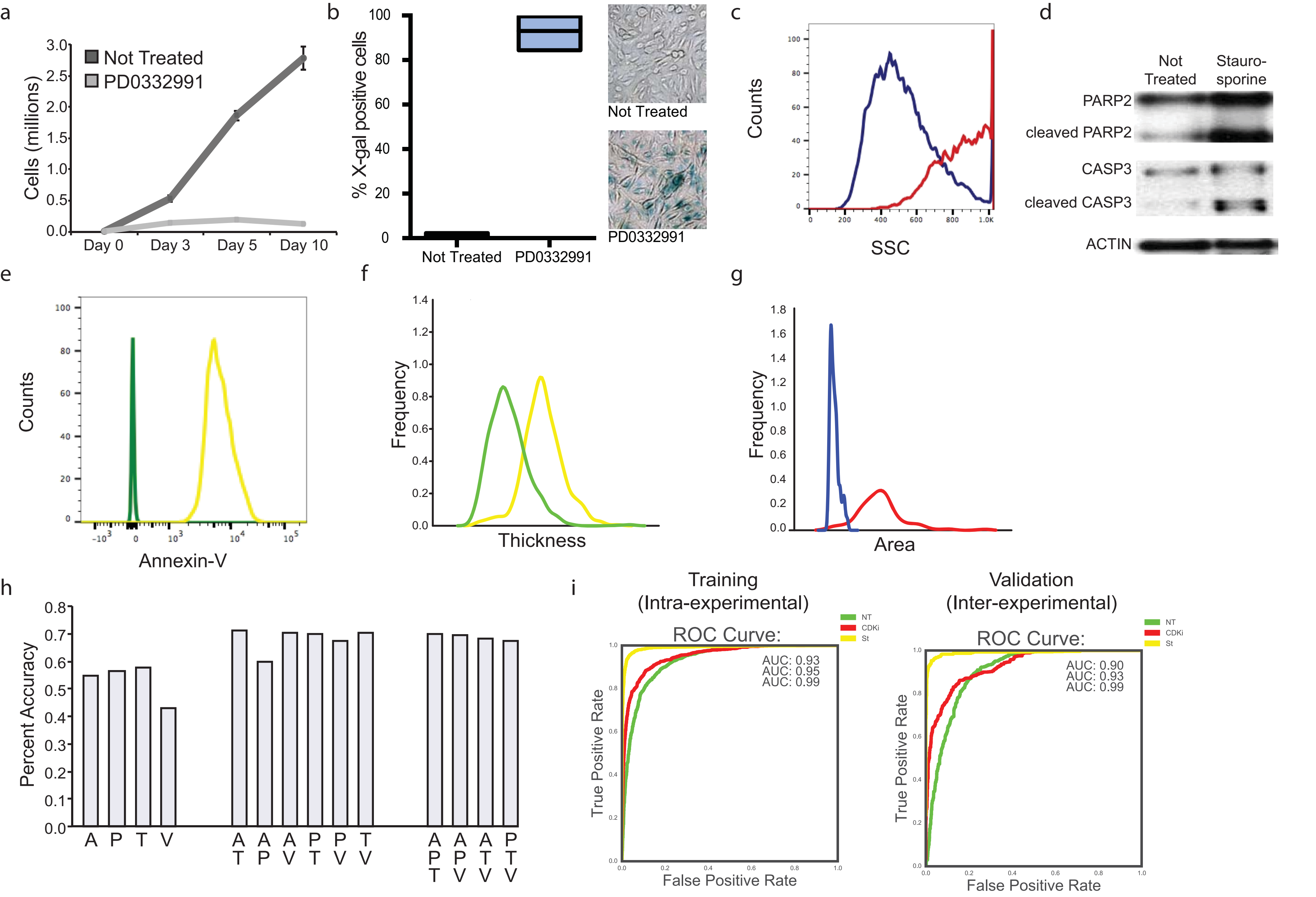

Supplemental Figure 1: a) Total number of cells for duration of imaging experiment plus one week (n=4, standard deviation of mean). b) Representative images and quantification of X-gal positive cells after 7 days treatment (n=5, mean and range). c) Senescence-associated side scatter (SSC) after 7 days PD0332991 (red) or control (blue) treatment (representative of 4 experiments). d) Western blot of indicated proteins 7 days after indicated treatment (representative of 4 experiments). e) Annexin-V expression by flow analysis 1 day after Staurosporine (yellow) or control (green) treatment (representative of 5 experiments). f) Histogram of DHC-derived thickness of cells analyzed in section e. g) Histogram of DHC-derived area of cells analyzed in section c. h) Percent accuracy of cell classification using each set of one, two or three features: area (A), perimeter (P), thickness (T) or volume (V). i) ROC curves for classifiers. The accuracy of a classifier trained on three independent experiments (left) does not diminish when applied to additional independent experiments (right).

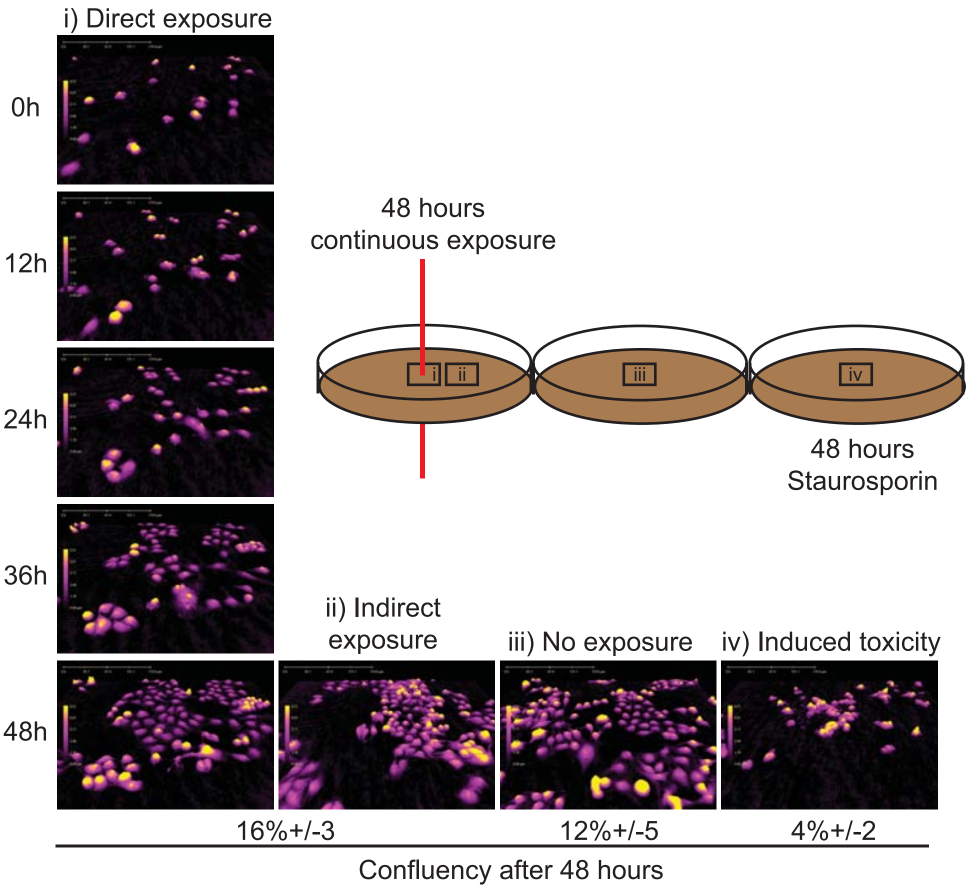

Supplemental Figure 2: Schematic, representative images, and quantification measuring phototoxicity from exposure to DHC laser. Per experiment, a single frame of A375 cells was exposed to the laser continuously for 48 hours. No cell death was observed during this time period. After 48 hours, the exposed cells were compared to cells within other frames of the same well (indirect exposure) and cells within other frames of adjacent wells (no exposure). No notable changes in morphology, nor changes in the confluency of exposed versus non-exposed wells were observed.

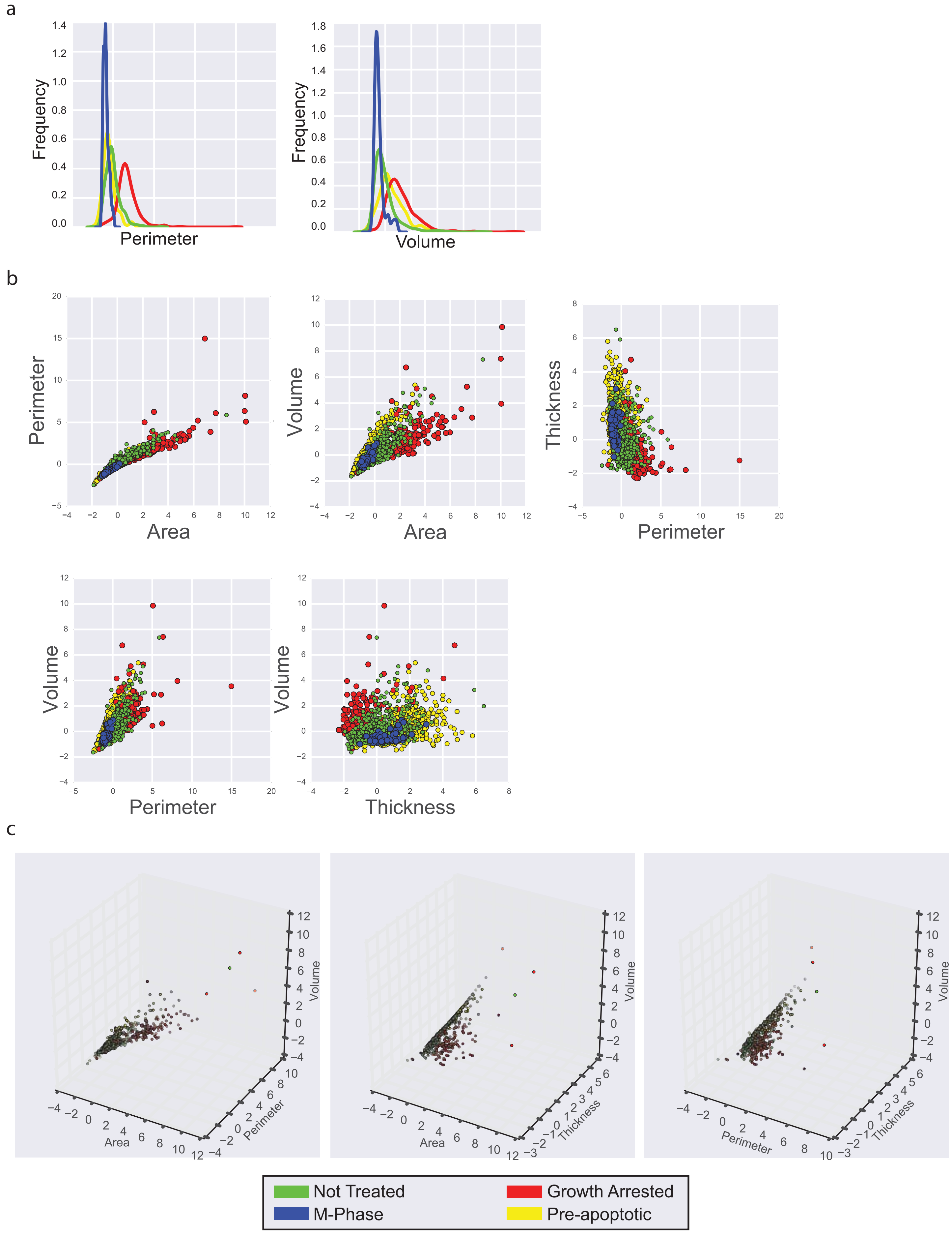

Supplemental Figure 3: 1D (a), 2D (b), and 3D (c) scatter plots of feature distribution for each cell state.

a

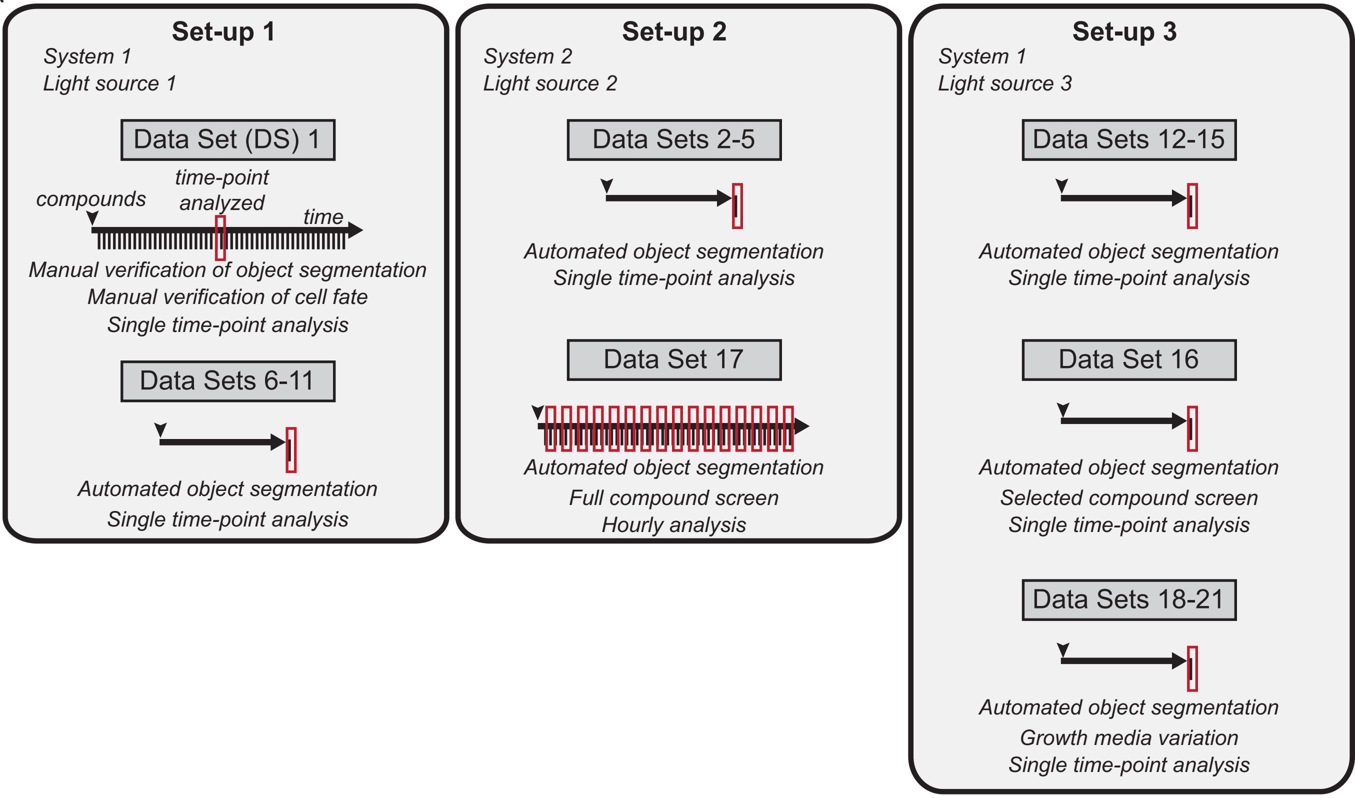

b

	Training sets	Application sets	
**Classifier 1**	Data Set 1	Data Set 1	*(Fig. 1i, S5)*
		Data Sets 2-11	*(Fig. 1j)*
		Background optimization	*(Fig. 2)*
**Classifier Series 2**	Subsets of 3 from Data Sets 2-11	Subsets from Data Sets 2-11 not used for training	*(Fig. S1i)*
**Classifier 3**	Data sets 2-11	Data sets 2-11	*(Fig. S6)*
		Data sets 12-15	*(Fig. S6)*
		Data set 16	*(Fig. S7)*
		Data set 17	*(Fig. 3)*
**Classifier Series 4**	Data Set 18-21	Data sets 18-21	*(Fig. S6)*

Supplemental Figure 4: a) Data sets generated in this study. Independent data sets were obtained with variable cell passage, media batches, and culturing plates over a time-span of two years. Different DHC set-ups were used, as indicated, with three independent light sources. In Data Sets 1-11, holograms were generated from technical duplicate wells and combined for each condition. Arrow heads represent addition of compounds, arrows represent time, vertical lines represent hologram acquisition, and red boxes indicate hologram analysis. b) Table of classifiers trained and applied in this study. Shown are data sets used to train the classifier and data sets to which the trained classifier was then applied.

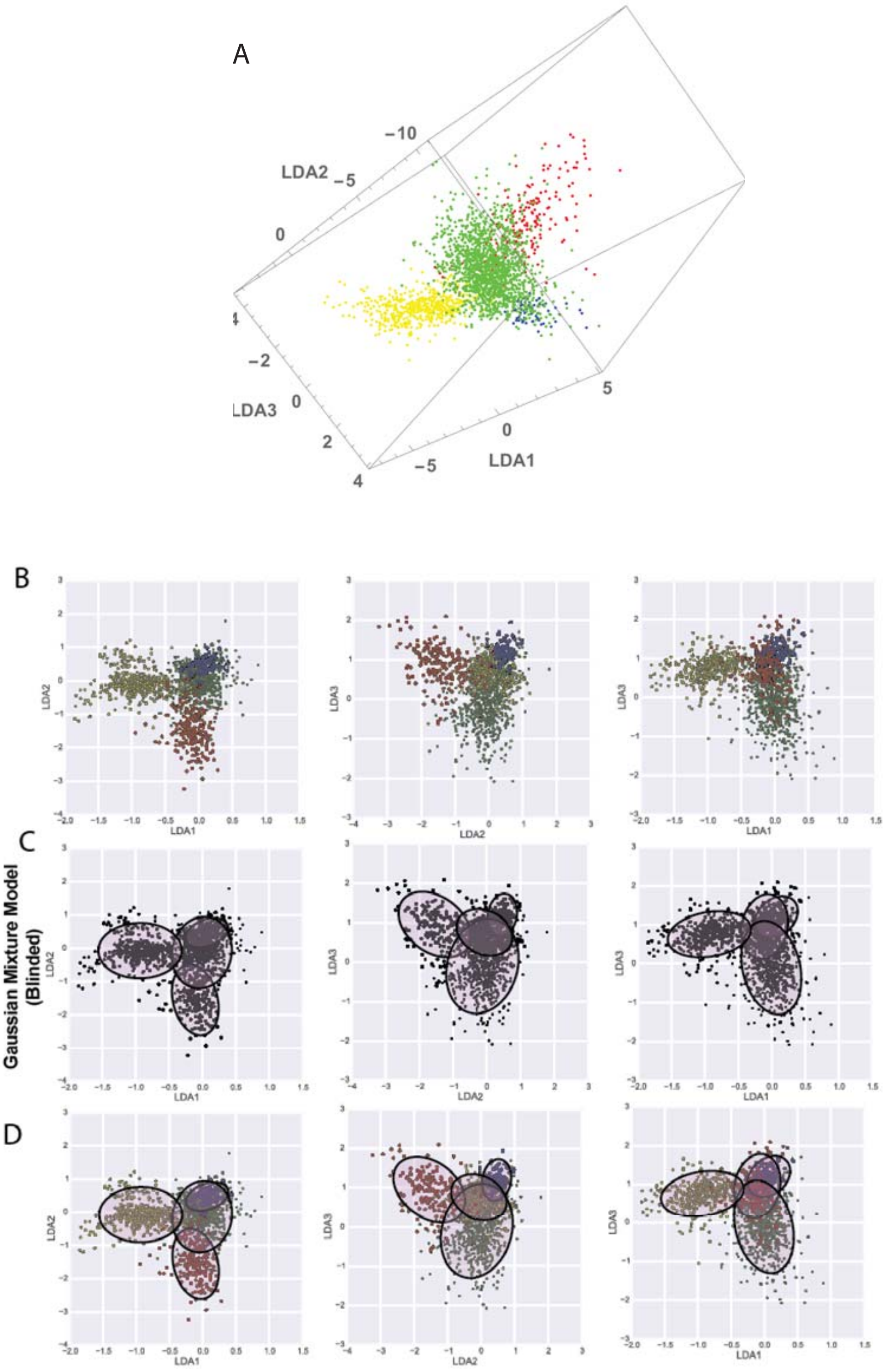

Supplemental Figure 5: a-b) Three-dimensional LDA space derived using twenty-six features, demonstrating clusters of pre-apoptotic (yellow), growth-arrested (red), non-treated (green) or M-phase (blue) cells. Data use 470 pre-apoptotic, 195 growth arrested, 66 M-phase, and 1527 non-treated cells. c) Four populations of cells identified by blinded Gaussian mix model of LDA space. Ellipses represent different views of three 3D predicted Gaussian distribution. d) Overlap of known cell states (dot colors) with predicted Gaussian distributions (ellipses).

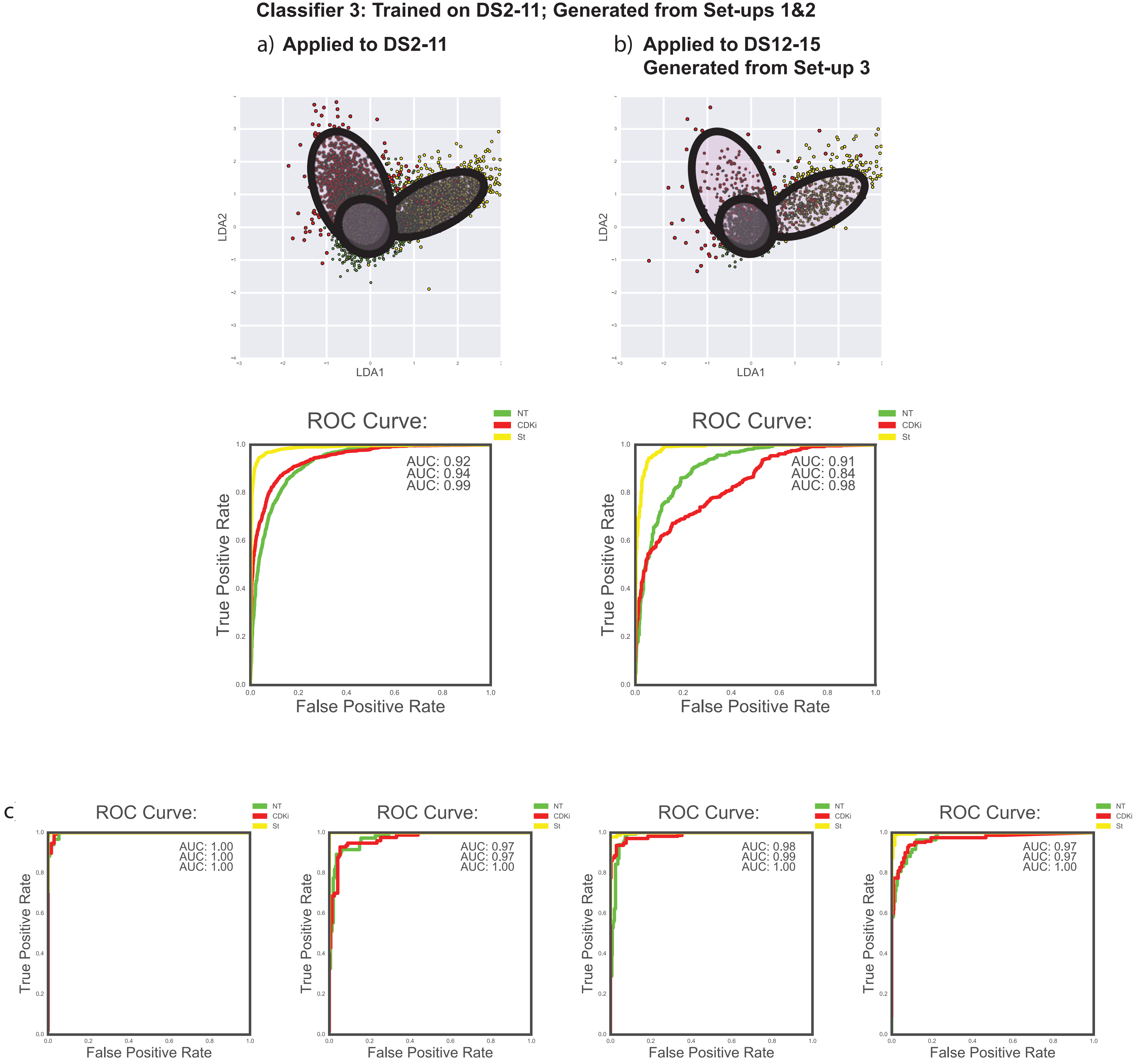

Supplemental Figure 6: a) LDA (top) and ROC curve (bottom) of Classifier 3 when applied to training set. Ellipses represent predicted Gaussian distributions of pre-apoptotic, growth-arrested, and normal cells in three-dimensional LDA space trained on DS2-11. Plotted are staurosporine (yellow), PD0332991 (red), and non-treated (green) cells from DS2-11. b) Classifier 3 applied to DS12-15, generated with independent set-up, plotted as in a). c) ROC curves showing accuracies of classifiers trained on and applied to cells grown in four different medias (independent lots of FBS and/or L-glutamate depletion).

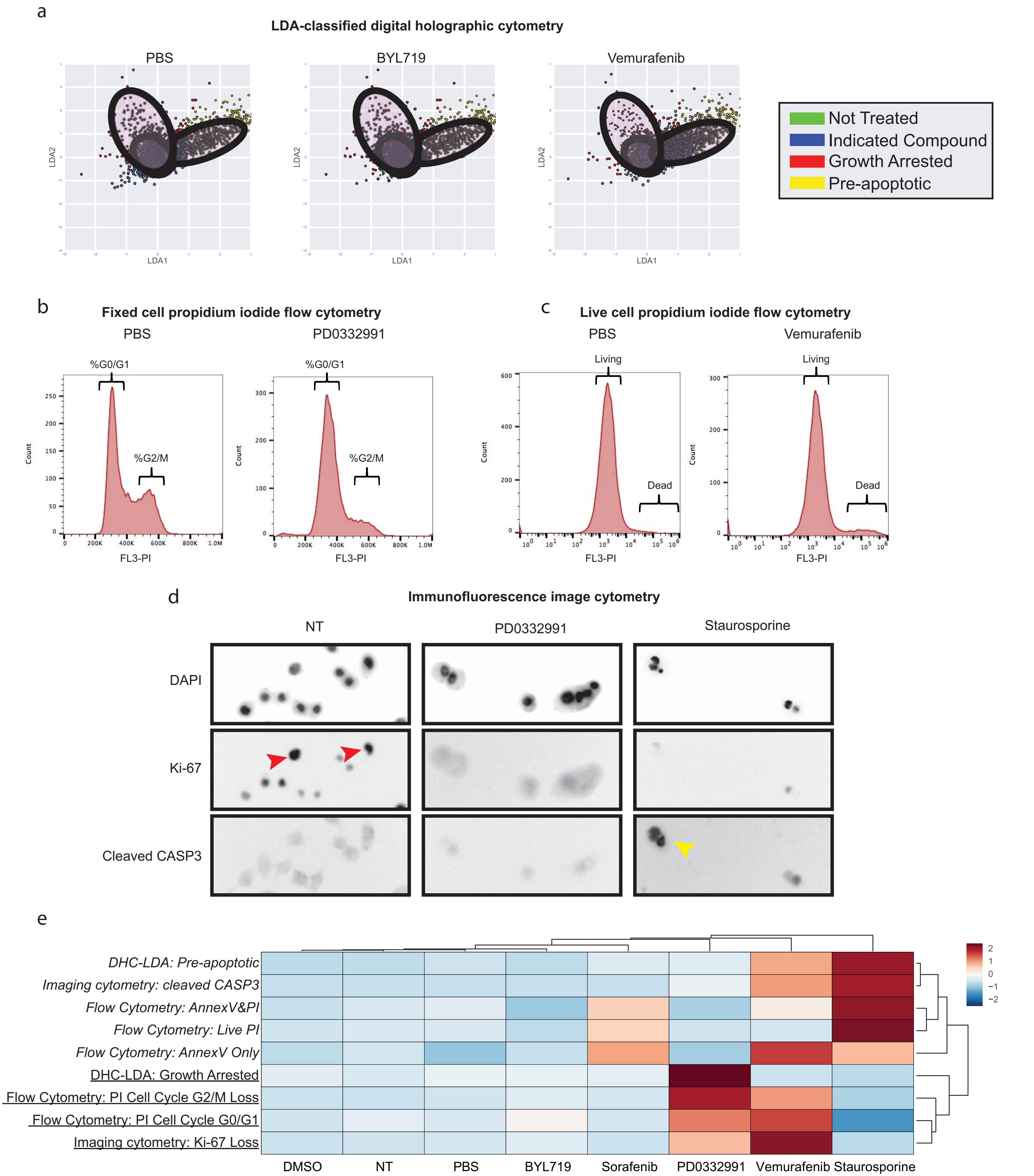

Supplemental Figure 7: a) Representative plots for DHC classification. Ellipses represent predicted Gaussian distributions of pre-apoptotic, growth-arrested, and normal cells in three-dimensional LDA space trained on previous data sets. Plotted are staurosporine (yellow), PD0332991 (red), indicated compound (blue), or non-treated (green) cells. Cells are designated pre-apoptotic or growth-arrested based upon location in LDA space. b-c) Representative plots of flow analyzed cell cycle (b) and cell death (c). d) Representative images of fluorescent imaging. Cells are designated as Ki-67 (red arrows) or cleaved CASP3 (yellow arrow) positive. e) Heatmap comparing normalized values of each assay.

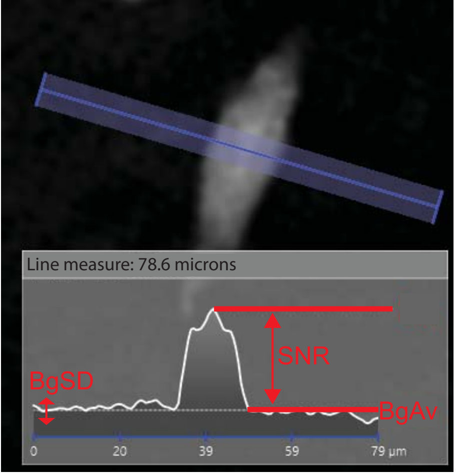

Supplemental Figure 8: Schematic of three potential hologram quality control (QC) metrics. Inset indicates intensity profile along blue line.

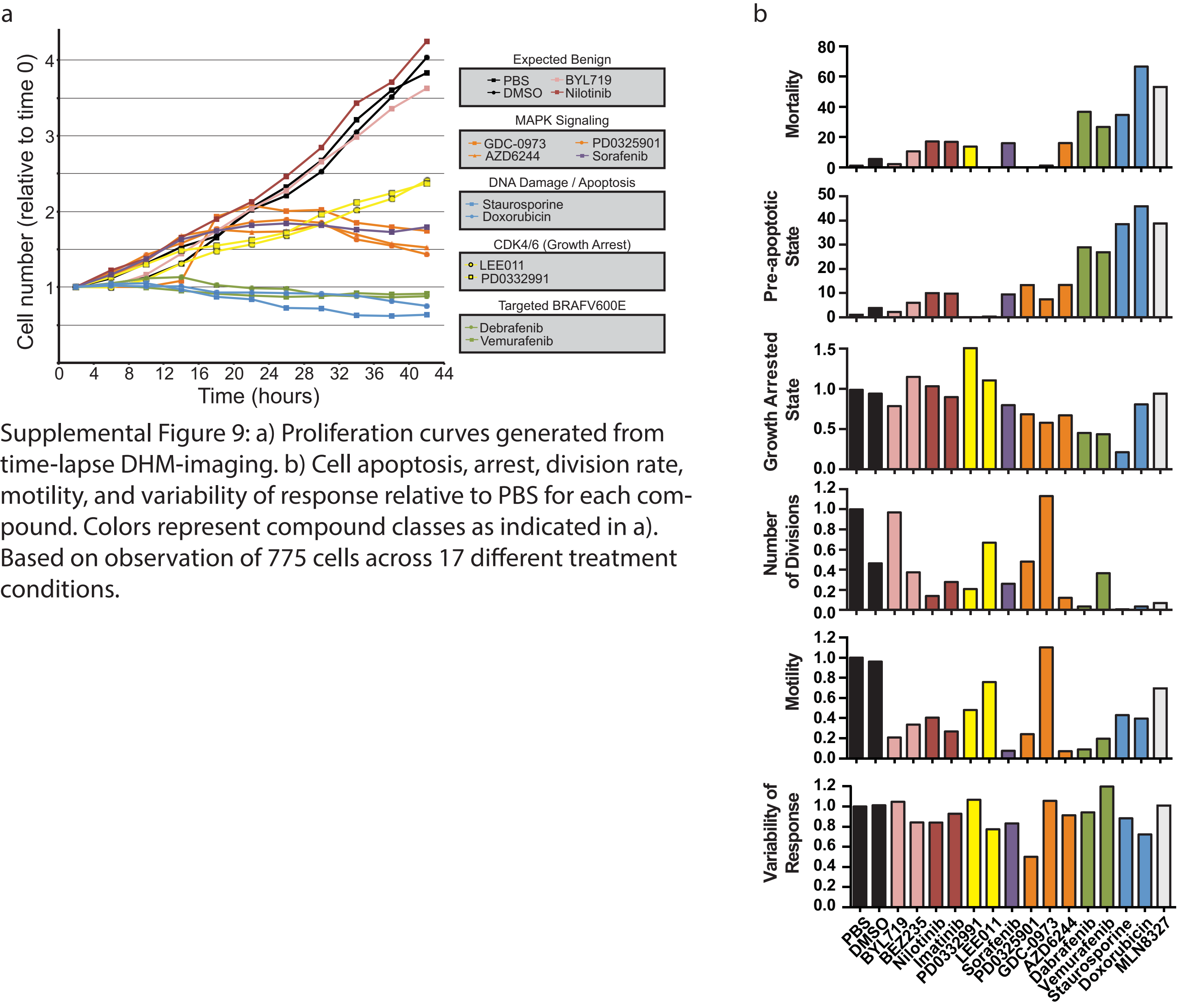

Supplemental Figure 9: a) Proliferation curves generated from time-lapse DHM-imaging. b) Cell apoptosis, arrest, division rate, motility, and variability of response relative to PBS for each compound. Colors represent compound classes as indicated in a). Based on observation of 775 cells across 17 different treatment conditions.

Supplemental Text 1: LDA Code

```
import math
import numpy as np
import scipy as spy
import scipy.stats as stats
import pandas as pd
from sklearn import metrics
from sklearn.metrics import roc_curve, auc, average_precision_score
from sklearn.metrics import precision_score, accuracy
from sklearn import linear_model
from sklearn.cross_validation import cross_val_score
from sklearn.cross_validation import train_test_split
from sklearn.preprocessing import label_binarize
from sklearn.lda import LDA
from sklearn import mixture
import matplotlib as mpl
import matplotlib.patches as mpatches
import matplotlib.pyplot as plt



UsedFeatures=[
 'Area (um2)',
 'Boxed breadth (um)',
 'Boxed length (um)',
 'Eccentricity',
 'Hull convexity',
 'Irregularity',
 'Perimeter length (um)',
 'Phaseshift min',
 'Phaseshift std. dev.',
 'Roughness avg',
 'Roughness kurtosis',
 'Roughness skewness',
 'Shape convexity',
 'Texture clustershade',
 'Texture clustertendency',
 'Texture contrast',
 'Texture correlation',
 'Texture correlation info1',
 'Texture correlation info2',
 'Texture energy',
 'Texture entropy',
 'Texture homogeneity',
 'Texture maxprob',
 'Thickness avg (um)',
 'Thickness max (um)',
 'Volume (um3)'];



FeaturesDataFrame= pd.DataFrame.from_csv(FeaturesDataFile);
ClassDataFrame= pd.DataFrame.from_csv(ClassDataFile);
UsedFeaturesDataFrame=FeaturesDataFrame[UsedFeatures];
```

```
UsedFeaturesClassDataFrame=pd.concat([UsedFeaturesDataFrame,ClassDataFr
ame],axis=1);

LDA_Features=UsedFeatures;
LDAFeaturesDataFrame,LDAClassDataFrame=[FeaturesDataFrame[LDA_Features]
,ClassDataFrame];

#LDA model data
Feature_Values=LDAFeaturesDataFrame.values;
Class_Values=LDAClassDataFrame[['Effector']].values.reshape(LDAClassDat
aFrame.shape[0]);
LDA_NComponents=3;

#Create LDA model
clf_lda = LDA(n_components=LDA_NComponents, priors=None, shrinkage=0.0,
solver='eigen',store_covariance=True, tol=0.00001);

#Fit LDA model
clf_lda.fit(Feature_Values,Class_Values);

#Transform Features to the LDA space
Feature_Values_Transformed=clf_lda.transform(Feature_Values);
Data_DataFrame=pd.concat([pd.DataFrame(Feature_Values_Transformed,index
=LDAFeaturesDataFrame.index,columns=['LDA'+str(i) for i in
range(LDA_NComponents)]),LDAClassDataFrame[['Effector']]],axis=1);


np.savetxt(Dir+'LDA_Data/lda_coord.dat',Feature_Values_Transformed,fmt=
"%f");
np.savetxt(Dir+'LDA_Data/lda_class.dat',Class_Values,fmt="%d");


LDA_axis_idx=[0,1];


#Define Gaussian Mixture Model
GMM_NComponents=3;
np.random.seed(36547207);

gmm =
mixture.GMM(n_components=GMM_NComponents,covariance_type='full',n_iter=
1000, n_init=5, tol=0.0001 ,random_state=seed);

#Train Gaussian Mixture Model
gmm.fit(Feature_Values_Transformed);

#Transform data to LDA space for plotting
Transformed_DataFrame=pd.DataFrame(clf_lda.transform(UsedFeaturesClassD
ataFrame
[LDA_Features].values),index=UsedFeaturesClassDataFrame[LDA_Features].i
ndex,columns=['LDA1','LDA2','LDA3']);
Transformed_DataFrameEffector=pd.concat([Transformed_DataFrame,ClassDat
aFrameAll[['Effector']]],axis=1);
Transformed_DataFrameEffectorDict={iEffector:Transformed_DataFrameEffec
tor.loc[Transformed_DataFrameEffector['Effector']==iEffector] for
iEffector in EffectorValuesList};
```

```
SizeDict={-1:50,0:20,1:40,2:30};
ColorsDataFrame=pd.DataFrame({'NT-mixed':[(0,1.0,0)],'NT - Actively
dividing':[(0,0,1.0)],'CDKinh':[(1.0,0,0)],'Staur':[(1.0,1.0,0)]},index
=['value']);

#Plot data in the LDA space

fig=plt.figure(figsize=(10, 10));
ax=fig.add_axes([0.2, 0.2, 0.8, 0.8]);
ax.set_xlabel("LDA"+str(LDA_axis_idx[0]+1),size=25),plt.ylabel("LDA"+st
r(LDA_axis_idx[1]+1),size=25,rotation=90);
LegendHandles = [mpatches.Patch(color=ColorsDataFrame['NT-
mixed']['value'], label='NT-mixed'),
mpatches.Patch(color=ColorsDataFrame['NT - Actively
dividing']['value'], label='NT - Actively
dividing'),mpatches.Patch(color=ColorsDataFrame['CDKinh']['value'],
label='CDKinh'),mpatches.Patch(color=ColorsDataFrame['Staur']['value'],
label='Staur')];
ax.legend(handles=LegendHandles,fontsize=12,loc=[1,1]);


#Plot components of the Gaussian Mixture Model
for iComp in range(GMM_NComponents):
    EVal, EVec =
np.linalg.eigh(gmm.covars_[iComp][LDA_axis_idx][:,LDA_axis_idx])
    EVec0_Norm,EVec1_Norm = [EVec[0]/np.linalg.norm(EVec[0]),
EVec[1]/np.linalg.norm(EVec[1])];
    angle = (np.pi+np.arctan2(EVec0_Norm[1], EVec0_Norm[0]))*180/np.pi
# convert to degrees
    Scale= 2.0;
    FaceColor=(1.0,0.0,1.0,0.15);
    EdgeColor=(0,0,0,1);
    ell = mpatches.Ellipse(gmm.means_[iComp][LDA_axis_idx],
2*np.sqrt(EVal[0])*Scale, 2*np.sqrt(EVal[1])*Scale, angle,
edgecolor=EdgeColor,
facecolor=FaceColor,antialiased=True,fill=True,linewidth=4,clip_box=ax.
bbox,axes=ax,label='Cluster '+str(iComp));
    ax.add_artist(ell);
    ax.annotate(str("%2.0f" % (gmm.weights_[iComp]*100.0))+'%',
xy=(gmm.means_[iComp][LDA_axis_idx[0]],gmm.means_[iComp][LDA_axis_idx[1
]]),fontsize=16,fontweight='bold',ha='center',va='center')


#Plot data scatter plot
for iEffector in [0,2,1]:

np.savetxt(Dir+'LDA_Data/lda_coord_'+str(iEffector)+'.dat',Data_DataFra
me.loc[Data_DataFrame['Effector']==iEffector][['LDA'+str(i) for i in
range(NComponents)]].values,fmt="%f");


    Data=Data_DataFrame.loc[Data_DataFrame['Effector']==iEffector];
    [Plot_data_x,Plot_data_y]=[Data.values[:,i] for i in LDA_axis_idx];
```

```
    Plot_data_xy = np.vstack([Plot_data_x,Plot_data_y]);
    Plot_data_z = np.ones(len(Plot_data_x));


    idx = np.lexsort((range(len(Plot_data_z)),Plot_data_z));


    [Plot_data_x,Plot_data_y,Plot_data_z] =
[Plot_data_x[idx],Plot_data_y[idx],Plot_data_z[idx]];
    offset=0.2;

Plot_data_z_scaled=(np.add(Plot_data_z,offset))/(max(Plot_data_z)+offse
t);


Plot_data_color=[tuple(tuple(ColorsDict[iEffector][k]*Plot_data_z_scale
d[i] for k in range(int(len(ColorsDict[iEffector]))))+(1.0,)) for i in
range(len(Plot_data_z_scaled))];
    Plot_data_size=[SizeDict[iEffector] for i in
range(len(EffectorVariable))];

    ax.scatter(Plot_data_x,
Plot_data_y,s=Plot_data_size,c=Plot_data_color);

plt.savefig("LDAPlot.pdf",format='pdf');
```
